# Integrin-mediated Signaling via Paxillin-GIT1-PIX Promotes Localized Rac Activation at the Leading Edge and Cell Migration

**DOI:** 10.7150/jca.32853

**Published:** 2020-01-01

**Authors:** Youjun Li, Xinzhou Wu, Zhiqiang Liu, Kanghui Lu, Rushi Liu, Xiangrong Guo

**Affiliations:** 1 Laboratory of Molecular and Cellular Biology, College of Life Sciences, Hunan Normal University, Changsha 410081, China; 2 Laboratory of Medical Molecular and Immunological Diagnostics, College of Medicine, Hunan Normal University, Changsha 410013, China

**Keywords:** integrin, localization Rac activation, leading edge, paxillin, signaling pathway

## Abstract

Rac activation is precisely regulated temporally and spatially by intracellular signaling pathways in migrating cells to guarantee the formation of specific cell protrusions-lamellipodia at the leading edge. Integrins-mediated adhesions also control the signaling pathway for localized Rac activation in the cells, but very few studies have been addressed in this field. In the study, we aim to focus on how integrin-mediated signaling affects localized Rac activation by reducing the paxillin expression with shRNA targeting paxillin. The results revealed that reduction of the paxillin expression in the cells inhibited the formation of focal adhesions and Rac activation. By using Rac FRET biosensor, Rac activation was localized at the leading edge of the cell, within the lamellipodium. A ternary complex of paxillin-GIT1-PIX could establish the signaling pathway in front of the cells. Thus, we described a mechanism of integrin-mediated signaling for localized Rac activation that upon ligand binding, activated integrin via the signaling pathway paxillin-GIT1-PIX promotes localized Rac activation at the leading edge and cell migration.

## Introduction

Activation of small GTPases, Rac or Cdc42 is initiated and regulated temporally and spatially by the integration and coordination of intracellular signaling when the trans-membrane receptors of the cell receive the stimuli from the extra-cellular matrix ligands. Activated or GTP-bound Rac or Cdc42 promotes actin polymerization and the protrusion formation, such as large, broad lamellipodia or spike-like filopodia at the leading edge of the cells, thus generating the driving force to push the cells forward. and leading to directional cell migration 1-6. There are two modes of mechanisms for regulating the activation of Rac or Cdc42 at the leading edge of migrating cells. During chemotaxix, phosphatidylinositol 3,4,5-triphosphate (PtdIns(3,4,5) P3) is locally accumulated at the leading edge, resulting in localized Rac activation to this edge^7^-^8^.. In the absence of chemo-attractants, the activated Cdc42 recruits PAR-6/PAR-3/aPKC complex to the front cell and orientates the microtubule-organizing center(MTOC) and Golgi apparatus in front of the nucleus^9^. Integrin-containing adhesions function as both structural and signaling ways in the cells, and the adhesions serve as the signaling centers orchestrating a network of signaling pathways including Rho GTPases^10^-^15^. Previous study established a mechanism of negative regulation of integrin-contained signaling for blocking Rac activation at the sides or in rear of the cells^16^, but integrin-mediated signaling for localized Rac activation in front or at the leading edge of the cells has not been elucidated. In the study, we focus on investigating how integrin-mediated signaling affects localized Rac activation at the leading edge by reducing the paxillin expression with shRNA targeting paxillin. The results revealed that reduction of the paxillin expression inhibited the formation of focal adhesions and Rac activation. By using Rac FRET biosensor, Rac activation was localized at the leading edge of the cell, within the lamellipodium. A ternary complex of paxillin-GIT1-PIX could establish the signaling pathway in front of the cells. Activated integrin via the signaling pathway paxillin-GIT1-PIX promotes localized Rac activation at the leading edge and cell migration.

## Materials and Methods

### Reagents and antibodies

Anti-Paxillin, anti-Rac, and fibronectin, purified from human plasma, were purchased from Millipore (Bedford, MA). Anti-GIT1, anti-βPIX were purchased from BD Biosciences. pEGFP-paxillin was purchased from Addgene.

### DNA expression constructs and ShRNAs to knockdown the paxillin expression

Human GIT1 cDNA was amplified by PCR with 5′ and 3′ primers incorporating *EcoR I* and *Sal I* restriction sites respectively, and was digested with *EcoR I* and *Sal I*, then cloned into pEGFP-N2 (Clontech, Palo Alto, CA) to generate pEGFP-GIT1. 3 plasmids of shRNA were designed and cloned by Shanghai JiKai Genechem to target the sequence of mouse paxillin ACTACATTTCAGCCCTCAA (1609- 1627 bp), TGAACTTGACCGGCTGTTA (581-599 bp) and TTAGATCAGTGGCAGCCTA (194-212 bp). To knockdown paxillin activity in the living cells the shRNA duplex was 5'-GATCCCGAACTACATTTCAGCCCTCAACTCGAGTTGAGGGCTGAAATGTAGTTCTTTTTGGAT-3' and 5'- AGCTATCCAAAAAGAACTACATTTCAGCCCTCAACTCGAGTTGAGGGCTGAAATGTAGTTCGG-3'; 5'-GATCCCtcTGAACTTGACCGGCTGTTACTCGAGTAACAGCCGGTCAAGTTCAGATTTTTGGAT and 5'- AGCTATCCAAAAATCTGAACTTGACCGGCTGTTACTCGAGTAACAGCCGGTCAAGTTCAGAGG-3'; 5'-GATCCCCTTTAGATCAGTGGCAGCCTACTCGAGAGGCTGCCACTGATCTAAAGTTTTTGGAT-3' and 5'- AGCTATCCAAAAACTTTAGATCAGTGGCAGCCTACTCGAGTAGGCTGCCACTGATCTAAAGGG-3' respectively, containing 2 stems and 1 loop structure. All the constructs were confirmed by DNA sequencing to ensure accuracy.

### Cell culture and transfection

Fibroblast CHO-K1 cells, were from American Type Culture Collection(ATCC) and maintained in DMEM medium supplemented with 10% fetal bovine serum (FBS). The cells, plated in 35-mm dishes and grown to confluence, were transfected with 2 μg of DNA and 4 μl of X-treme Gene HP (Roche, Indianapolis, IN), or co-transfeced with 1 μg of each DNA and 4 μl of X-treme Gene HP following the manufacturer's instructions. The cells were used 18-24 h post transfection.

### Immunoprecipitation,western blotting and Rac activation assay

At 18 h after transfection, the cells were detached and plated on 5µg/ml fibronectin-coated 35-mm dishes and incubated at 37°C for the indicated time. The cells were lysed in lysis buffer (50 mM Tris-HCl pH 7.5, 150 mM NaCl, 1 mM EDTA, 0.1% SDS, 1% Triton X-100 plus halt protease inhibitor cocktail and halt phosphatase inhibitor cocktail). For western blotting, the lysed cells were incubated with 2 μg of monoclonal anti-GIT1 Ab at 4°C with shaking for 2 h. The immunoprecipitates were captured with 20 μl of protein G-Sepharose beads (Millipore) on ice overnight. For Rac activation assay, the lysed cells were incubated with 2 μg of PAK-PBD beads(Millipore) at 4°C with shaking for 2 h to capture GTP-bound Rac. The whole cell lysates were also assayed to probe total Rac content. the beads were washed three times with lysis buffer and clarified by centrifugation. The proteins were denatured in SDS sample buffer by boiling water, and protein concentrations were determined using Bradford method to ensure equal amounts of total protein (30 ng) were loaded in each well. The protein samples were resolved on 10% SDS-PAGE and transferred to PVDF. Western blots were visualized and imaged with McAb anti-paxillin, anti-βPIX or anti- Rac using Super-Signal West Pico (Pierce) under ChemiDoc TM XRS (Bio-rad), and quantified as integrated density to represent the amount of proteins with Image J software.

### Immuno-fluorescence and Rac FRET assay

For immuno-fluorescence assay, the cells were grown on fibronectin-coated MatTek dishes and transfected. At 18h after transfection, the cells were fixed with 4% paraformaldehyde in PBS and stained with monoclonal anti-paxillin Ab and FITC- conjugated goat-anti-mouse IgG as standard procedure. For co-localization of paxillin with GIT1, or GIT1 with PIX, the cells were transfected with pEGFP-paxillin or pEGFP-GIT1, and fixed, stained with anti-GIT1, or anti-βPIX Ab, and TRITC- conjugated goat-anti-mouse IgG. The intra-molecular biosensor for Rac activation, namely GPR, was designed based on fluorescence resonance energy transfer (FRET) and described previously^6^. In construct,the biosensor of GPR contains EGFP, binding domain of PAK, RAC and DsRed sequentially from N-terminus to C-terminus. The cells were co-transfected with negative control and Rac biosensor GPR or shRNA and GPR. At 18 h after transfection, the cells were detached with trypsin and plated on 5 μg/ml fibronectin-coated MatTek dishes and incubated at 37 °C for 5min, and fixed. For FRET assay *in situ*, the cells grown on MatTek dishes were co-transfected with negative control and GPR or shRNA t and GPR. At 18 h after transfection, 5μg /ml of fibronection was added to the medium and the cells were incubated at 37°C for 5 min, and fixed. For sensitized emission, GFP was excited by using the argon 488 nm laser line, and detected with emission filter 515/30 for GFP/GFP. For FRET measurement, FRET imaging was acquired with excitation argon 488 laser line and emission filter 590/70 for DsRed/GFP. All the images were viewed on a Nikon two-photon laser scanning confocal A1+microscope, and fluorescence intensities of the FRET signals were measured with the software equipped on the microscope.

### Transwell assay

Transwell assay was performed using 24 well transwell permeable supports with 8 μm pores (Corning) coated with fibronectin (5μg /ml) and placed over a bottom chamber containing 10% FBS DMEM according to the manufacturer's instructions 17. Briefly, at 12h after transfection, the cells expressing EGFP were collected and counted by sorting EGFP-positive cells in a BD FACSAria III cell sorter with 488-nm laser. The transfected cells were added to the filter, serum-starved for 12 h and placed over the bottom chamber. After 12h incubation at 37 °C, the cells that migrated to the bottom chamber were collected and counted by flow cytometry. The samples were assayed in triplicate.

## Results

### shRNA targeting paxillin efficiently inhibited the formation of focal adhesions by reducing the expression of paxillin

**Figure 1:**
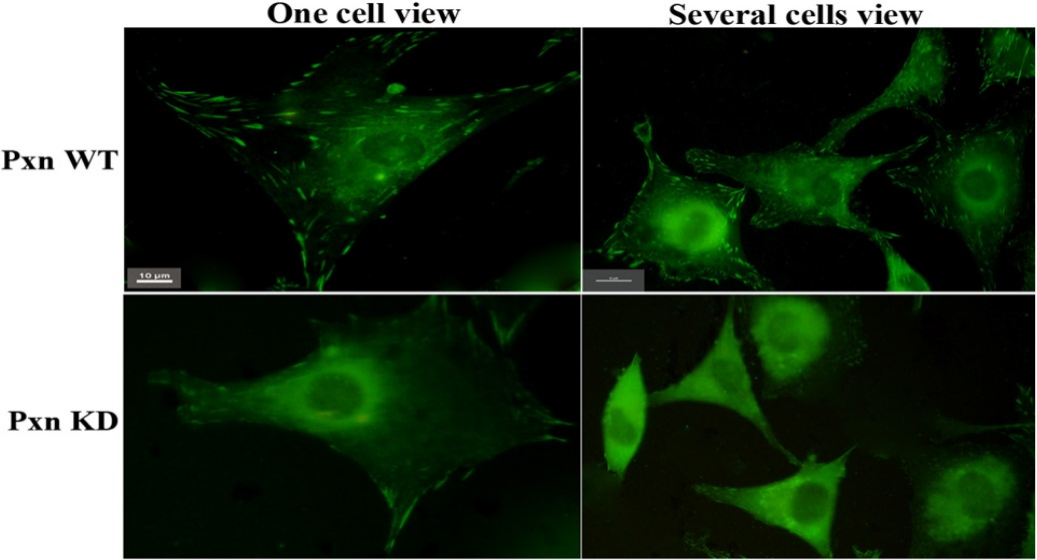
** Many more focal adhesions were observed in the transfected cells with negative control (Pxn WT )than with shRNA targeting paxillin(Pxn KD).** CHO cells grown on MatTek dishes were transfected with negative control or shRNA targeting paxillin. At 18 h after transfection, 5µg /ml of fibronectin was added to the medium and the cells were incubated at 37°C for 5 min. The cells were fixed, stained with anti-paxillin McAb and FITC-conjugated goat-anti-mouse IgG. Upper panel: transfected cells with negative control (Pxn WT). Lower panel : transfected cells with shRNA targeting paxillin(Pxn KD). Scale bar 10µm

To ascertain the effect of integrin-mediated signaling through paxillin on Rac activation. 3 shRNAs targeting paxillin were designed and performed to knockdown paxillin activity in the cells. Fig.1 clearly showed that much more focal adhesions were observed in the cells transfected with negative control(paxillin WT) than in the cells with shRNA targeting 1609-1627 bp of paxillin (paxillin Knockdown) , indicating that the shRNA efficiently inhibited the formation of focal adhesions by reducing the expression of paxillin in the cells.

### Reduction of the paxillin expression inhibited adhesion-induced Rac activation, and Rac activation was localized at the leading edge of the cells

**Figure 2:**
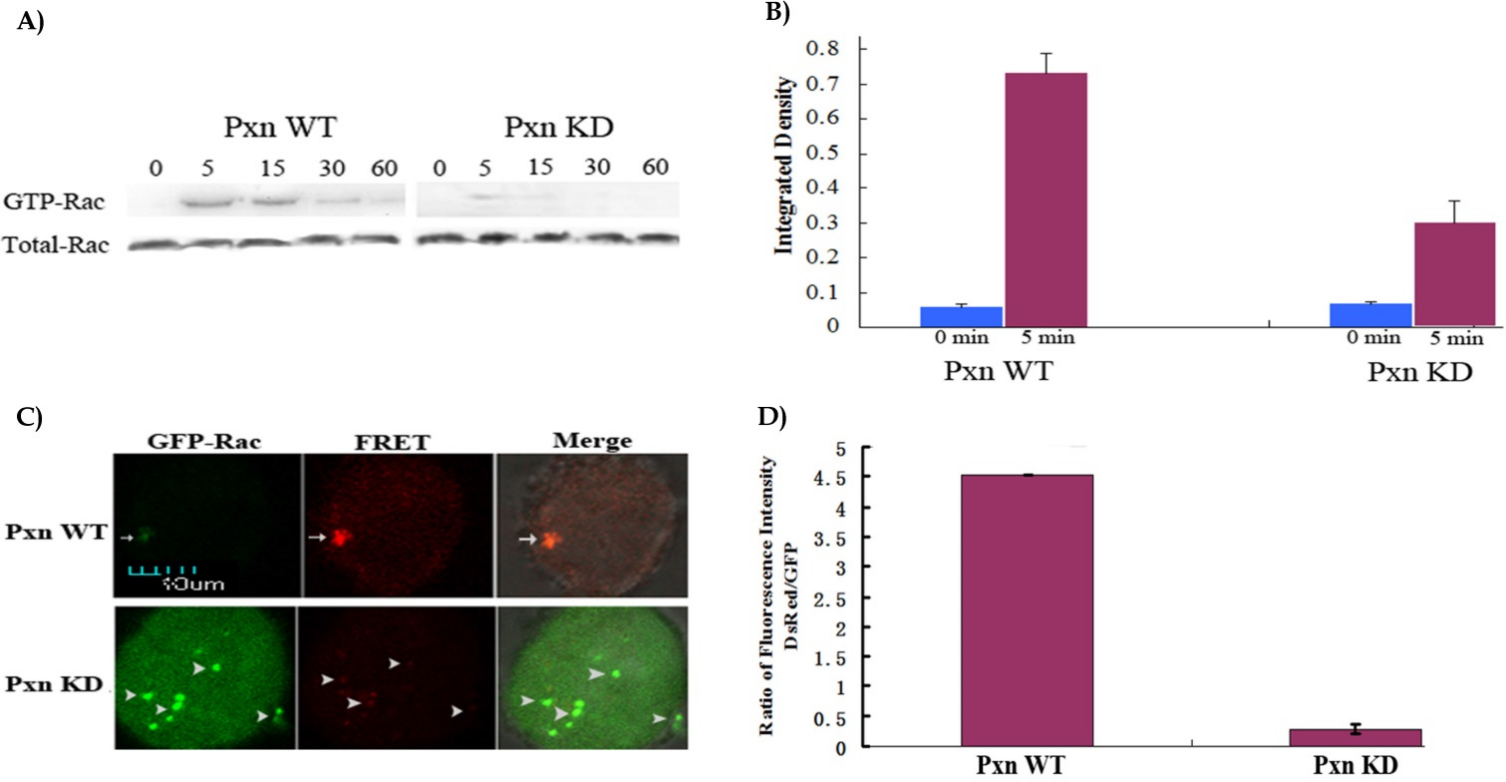
** Reduction of paxillin expression inhibited Rac activation in the cells. A)** CHO cells grown in 35mm dishes were transfected with negative control or shRNA targeting paxillin. At 18 h after transfection, the cells were detached with trypsin and plated on 5 µg/ml fibronectin-coated dishes and incubated at 37 °C for the indicated durations. Cell lysates were incubated with immobilized p21-binding domain of PAK (GST-PBD) beads. The GTP bound form of Rac was detected by western blotting and indicated as GTP-bound Rac (upper panels). Whole cell lysates were also probed to assay total Rac content (lower panels). **B)** GTP-bound Rac and total Rac(Fig. 2A) in transfected cells at 0 and 5 min adhering to fibronectin were quantified as integrated density using Image J to represent the amount of protein. The vertical axis shows the quantified data as normalized to total Rac. **C)** CHO cells were co-transfected with negative control and Rac biosensor GPR or shRNA targeting paxillin and GPR. At 18 h after transfection, the cells were detached with trypsin and plated on 5 μg/ml fibronectin-coated MatTek dishes and incubated at 37°C for 5min, and fixed. GFP was excited by using the argon argon 488 nm laser line, and detected with emission filter 515/30 for GFP/GFP. For FRET measurement, FRET imaging was acquired with excitation argon 488 laser line and emission filter 590/70 for DsRed /GFP. All the images were viewed on a Nikon two-photon laser scanning confocal A1+microscope. Upper panel: Arrows indicate positive FRET signals showing that Rac was activated in paxillin WTcells. Lower panel: Arrowheads indicate no FRET signals showing that Rac was not activated in paxillin KD cells. Scale bar 10μm. **D)** The ratio of fluorescence intensity of DsRed to GFP for one positive FRET signal was much higher than that eight negative FRET signals(Fig. 2C). The fluorescence intensity of DsRed and GFP for FRET signals was measured with the software equipped on a Nikon two-photon laser scanning confocal A1+microscope.

When the expression of paxillin was efficiently reduced in the cells by shRNA, next we performed western blotting to identify the effect of integrin-mediated signaling through paxillin on Rac activation. As shown in Fig.2A,2B, GTP-Rac was higher after the paxillin WT cells were adherent to fibronectin for 5 min, and the signal was still visible at 60 min. In contrast, GTP-Rac in paxillin KD cells was only 38.9%(0.28/0.72) of that in paxillin WT cells after adhesion to fibronectin for 5 min, and almost no signal was detected at other indicated durations. 5 min was regarded as the duration in the cells adhering to fibronectin to induce Rac activation by activated integrin. The result indicated that reduction of the paxillin expression inhibited adhesion-induced Rac activation in the cells.

To visualize and image Rac activation in the cells, Fluorescence resonance energy transfer(FRET) assay using the specially-designed biosensor with the donor GFP and the acceptor DsRed was performed 6. Fig. 2C depicted that FRET occurred in the cells co-transfected with GPR and negative control. The binding together of activated Rac and its effector PAK brought about the binding together of two fluorescent proteins, GFP and DsRed: Very weak green fluorescence appeared in the imaged cell when the biosensor was excited with 488 nm laser line and detected with emission filter 515/30 for GFP/GFP, but strong red fluorescence appeared in the cell when the biosensor was excited with 488 nm laser line and detected with emission filter 590/70 for DsRed /GFP, indicating that 508 nm laser, which was emitted from GFP, was strong enough to excite DsRed to emit 583 nm laser(red fluorescence). In contrast, no FRET was found in the cells co-transfected with GPR and shRNA. Rac was not activated, and not bound to PAK, leading to no space conformational change of GFP and DsRed: Strong green fluorescence was viewed in the cell for GFP/GFP, and no red fluorescence was viewed in the cell for DsRed /GFP, indicating that 508 nm laser was emitted off from GFP(green fluorescence). Fig. 2D showed that the ratio of fluorescence intensity of DsRed to GFP was 4.52(3740/828) for one positive FRET signal, 0.28(1138/4095) for eight negative FRET signals respectively. The positive and negative FRET signals varied greatly in the ratio. FRET assay confirmed that reduction of the paxillin expression inhibited Rac activation. focal adhesions coincided with Rac activation.

**Figure 3:**
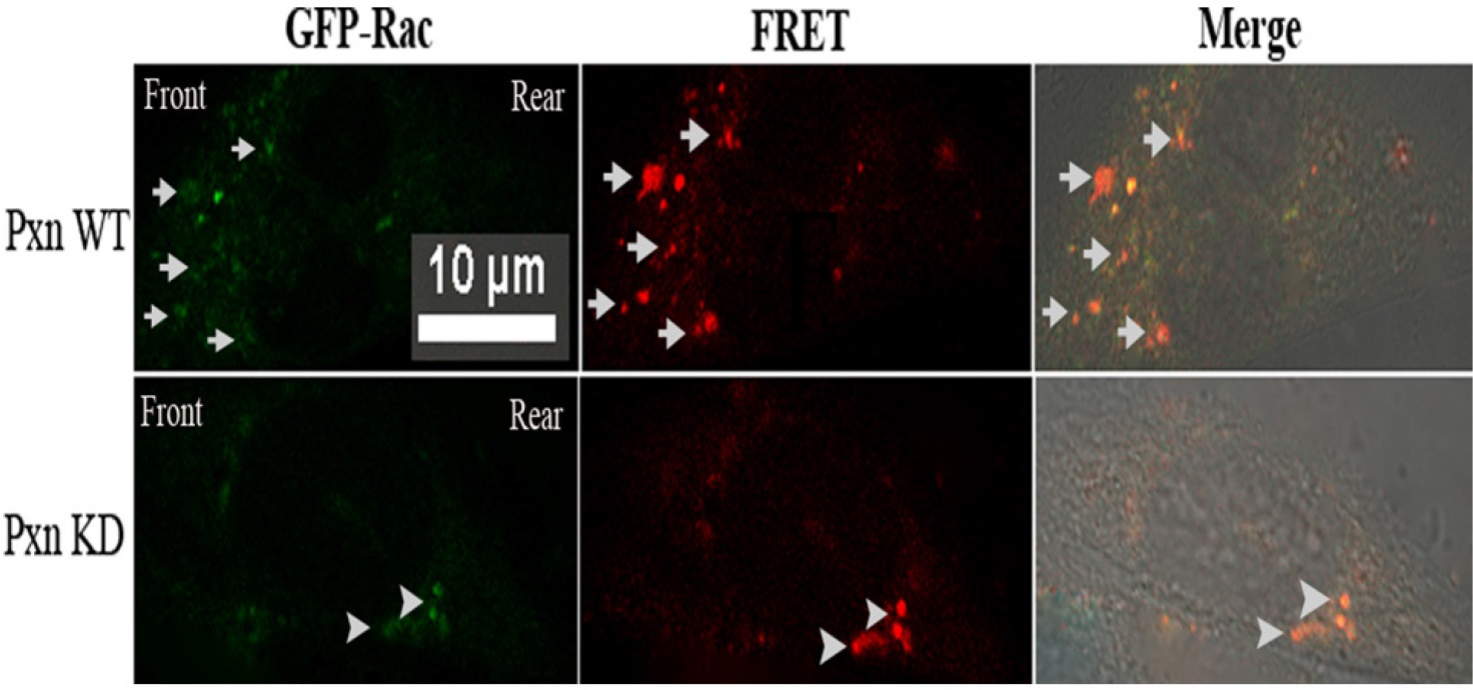
** Rac FRET assay *in situ* in paxillin WT and KD cells respectively revealed that many positive FRET signals were found within lamelliopodium in paxillin WT cells, indicating that Rac was activated at the leading edge. In contrast, no such positive FRET signals were found within the lamellipodium in paxillin KD cells, but only several stronger FRET signals appeared in the rear of the cell.** CHO cells grown on MatTek dishes were co-transfected with negative control and Rac biosensor GPR or shRNA targeting paxillin and GPR. At 18 h after transfection, 5µg /ml of fibronection was added to the medium and the cells were incubated at 37°C for 5 min, and fixed. GFP and FRET measurement were the same as Fig. 2C. Scale bar 10µm. Upper panel: Arrows indicate positive FRET signals at leading edge within lamelliopodium of paxillin WT cells. Lower panel: Arrowheads indicate stronger FRET signals appeared in the rear of paxillin KD cells.

It was difficult to discern the cell polarity from the round-shaped cells detached with trypsin. To ascertain the localized Rac activation in the cells. fibronectin was added to the medium of MatTek dishes at 18 h after transfection and the cells were incubated at 37°C for 5 min, and Rac FRET assay *in situ* was performed in paxillin WT and KD cells respectively. Fig 3.depicted that in paxillin WT cells, FRET indeed occurred in front of the imaged cell: many locally activated Rac signals appeared at the leading edge, and no such signals were detected in rear or at two sides of the cell, indicating that activated Rac was localized at the leading edge, within the lamellipodium. In contrast, no such positive FRET signals were found within the lamellipodium in paxillin KD cells, but only several stronger FRET signals appeared in the rear of the cell.

### The ternary complex of paxillin-GIT1-PIX residing in front of the cell could establish the physical basis for the signaling pathway at the leading edge of the cells, and promoted cell migration

**Figure 4:**
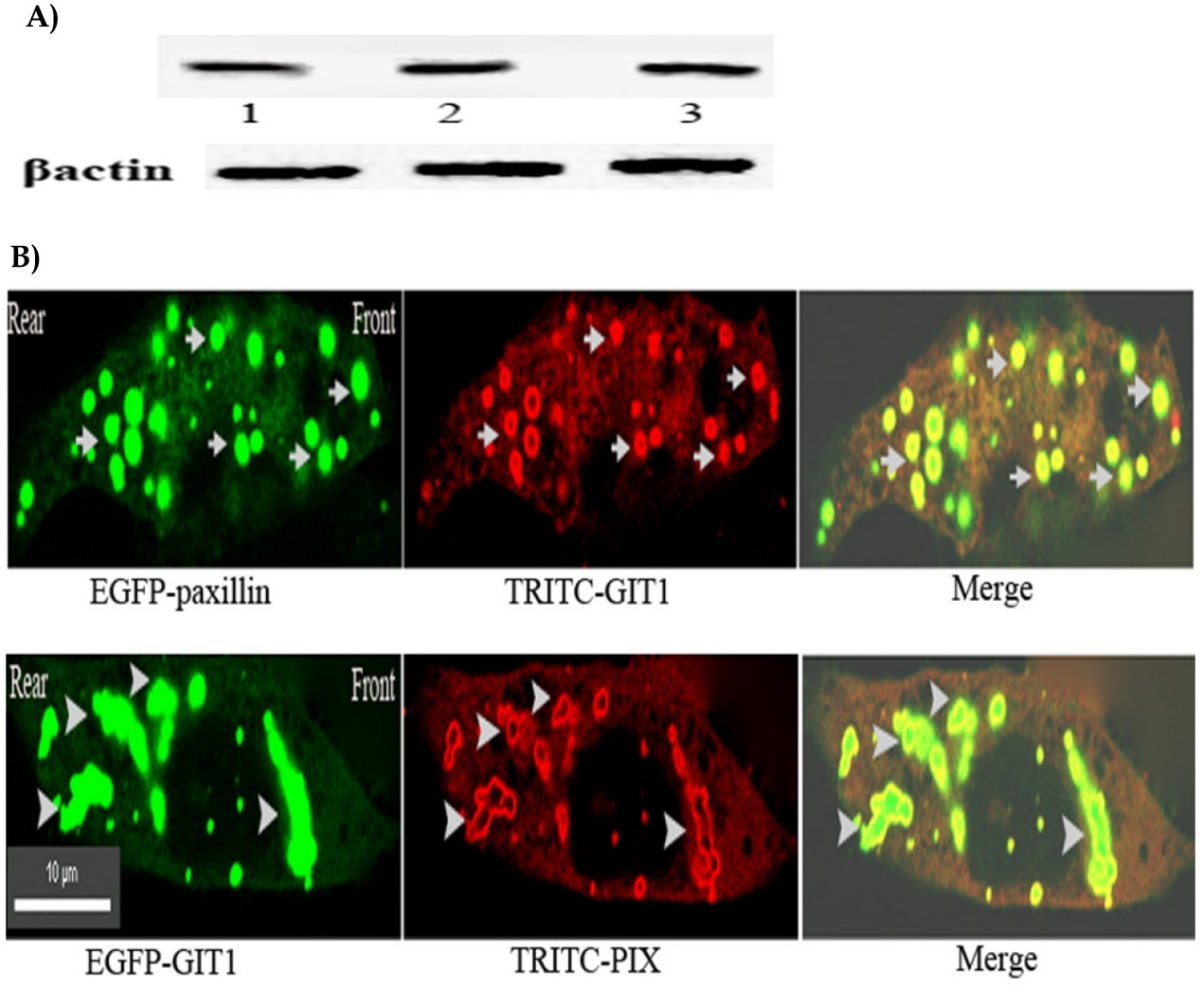
** A) GIT1 possessed the strong physical interaction with paxillin and PIX.** CHO cells were detached with trypsin and plated on 5 μg/ml fibronectin-coated dishes and incubated at 37 °C for 5 min. Cell lysates were incubated with anti-GIT1 McAb to collect the protein complex. Paxillin or PIX of the protein complex was detected by western blotting using anti-paxillin McAb or anti- β PIX McAb. Upper panel:1. IP:GIT1+WB:GIT1, 2. IP:GIT1+WB:paxillin, 3. IP:GIT1+WB:PIX. Lower panel: β-actin loading control. **B) GIT1 was co-localized with paxillin and PIX in front or rear of the nucleus of the cell.** CHO cells were transfected with pEGFP-paxillin or pEGFP-GIT1. At 18 h after transfection, 5μg /ml of fibronectin was added to the medium and the cells were incubated at 37^。^C for 5 min, and fixed. stained with anti-GIT1, or anti- β PIX McAb, and TRITC-conjugated goat-anti-mouse IgG. All the images were viewed on a Nikon two-photon laser scanning confocal A1+microscope. Upper panel: Arrows indicate the co-localization of GIT1 with paxillin in front or rear of the neucleus of the cell. Lower panel: Arrowheads indicate the co-localization of GIT1 with PIX in front or rear of the neucleus of the cell. Scale bar 10µm.

The results mentioned above confirmed that activated integrin stimulated Rac activation at the leading edge of the cells through paxillin, but as a cytoplasmic adaptor protein of focal adhesions, paxillin is not physically associated with Rac. To identify how the activated integrin transmits the signaling from integrin to Rac, the possible signaling pathway from integrin to Rac is paxillin-GIT1-PIX. GIT1, which is involved in many cell processes and possesses multi-protein binding domains, has both PBD domain combining with paxillin and SHD domain connecting with PIX, a PAK-interacting exchange factor for Rac, is an ideal intermediate component of the signaling pathway through paxillin to Rac^18^-^24^. To test the possibility, we investigated the physical interactions of GIT1 with paxillin and PIX by using anti-GIT1 McAb in Immunoprecipitation to collect the protein complex, and using anti-paxillin or anti-βPIX McAb in western blotting respectively to detect paxillin or PIX in the protein complex. Fig 4A. clearly showed that GIT1, a multifunctional protein, displayed a strong physical interaction with paxillin and PIX, indicating that a ternary complex of paxillin-GIT1-PIX could exist in the cells.

To further ascertain the spatial distribution of the protein complex in the cells. Immuno-fluorescent co-localization was performed. Fig.4B. illustrated that GIT1 was mostly co-localized with paxillin and PIX in front or rear of the nucleus of the cells. Taken together, the results confirmed that the ternary complex of paxillin-GIT1-PIX residing in front of the cell could establish the physical basis for the signaling pathway at the leading edge of the cells.

**Figure 5:**
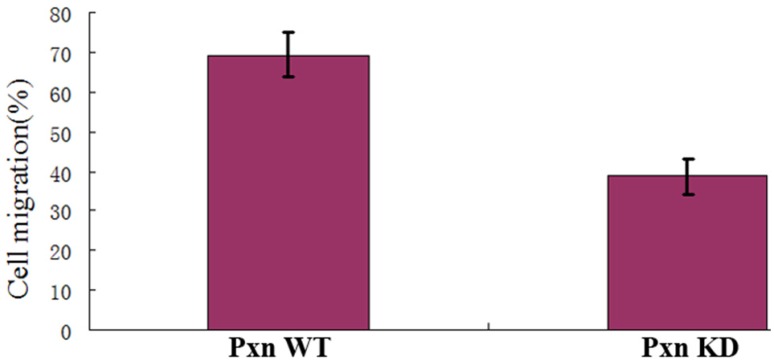
** Integrin-mediated signaling for localized Rac activation promoted cell migration.** The vertical axis shows the cell rates of the cells that migrated to the bottom chamber to the whole cells in transfected cells with negative control or shRNA in transwell assays (P<0.01).

To identify the real effect of integrin-mediated signaling pathway for activating Rac on cell migration, the transwell assay was performed.As shown in Fig. 5, the rate of the cells that migrated to the bottom chamber was 69.4% for the paxillin WT cells and 38.6% for paxillin KD cells respectively. The data indicated that integrin-mediated signaling pathway promoted Rac activation and thereby promoted cell migration.

## Discussion

Cell migration begins from the integration of several dynamic processes, including the formation of specific protrusions, and assembly and disassembly of focal adhesions^25^. These processes maintain and stabilize each other to establish the linkage between extra-cellular matrix (ECM) and actin cytoskeleton^12^,^18^. Activated Rac plays the pivotal role in regulation of actin polymerization and lamellipodium formation at the leading edge of the cells, thus, cell polarity, or directional cell migration depends on localized Rac activation^6^,^16^. Previous study established a mechanism of negative regulation of Rac activation at the sides or in rear of the cells^16^. Restriction of α4 integrin phosphorylation to the leading edge limits the interaction of α4 with paxillin to the sides and rear of a migrating cell^16^,^26^,^27^. The α4-paxillin association at the sides or the rear of the cell formed a ternary complex by recruiting an ADP-ribosylation factor GTPase activating protein (Arf-GAP) that inhibits Rac activity, and the complex formed a signaling pathway α4-paxillin-Arf-GAP and blocked Rac activation^16^, but how integrin-mediated signaling affects localized Rac activation at the leading edge has not been elucidated. In the study, we investigated the signaling from integrin to Rac through paxillin by reducing the paxillin expression with shRNA targeting paxillin. The results reveal that reduction of the paxillin expression efficiently inhibited the formation of focal adhesions and Rac activation. High coincidence of focal adhesions with Rac activations reveals and supports that Rac, or Rho GTPases, are regulated by signaling networks in adhesions, and in turn, adhesion assembly is regulated by Rho GTPases^12^. It is required to adopt an ideal biosensor to ascertain the localized activation in the cells. Rac FRET assay with the specially- designed biosensor allowed us to quantitatively visualize and image Rac activation with high spatial and temporal resolution in cellular context with a confocal microscope^6^. We can easily identified activated Rac by just viewing FRET signals and calculating the ratio of fluorescence intensity of the acceptor to donor. When FRET occurs, the donor emission (green fluorescence) is decreased or quenched, but the acceptor emission(red fluorescence) is activated or increased. Rac FRET assay *in situ* confirmed that Rac activation was localized at the leading edge, within the lamellipodium.

Our result showed that GIT1 possessed strong physical interactions with paxillin and PIX. GIT1 was co-localized with paxillin and PIX in front or rear of the nucleus, indicating that a ternary complex of paxillin-GIT1-PIX could establish the signaling pathway in front of the cells. Because GIT1 was bound toα4 subunit only in the presence of paxillin^16^, paxillin joined the complex before GIT1. The signaling from integrin was through paxillin to GIT1. Taken all the results together, we can describe a mechanism of positive regulation of integrin-mediated signaling for localized Rac activation: at the leading edge of migrating cells, upon ligand binding, activated integrin, which is phosphorylated at 988S, and no physical association with paxillin^16^, induces paxillin to recruit GIT1, and GIT1 interacts with PIX. PIX, as an exchange factor of Rac, recruits PAK and activates Rac activity. Activated Rac initiates the signaling pathways by combining with PAK, leading to actin polymerization and lamellipodium formation, and thus promotes cell migration^2^. lntegrin-containing adhesions are highly transient and localized, and facilitate temporal and spatial activation of the signaling pathway^12^,^28^, and thereby drive directional cell migration.
